# Supplementary material for: The genome sequence of the barkfly, Psococerastis gibbosa (Sulzer, 1776)
Source: Wellcome Open Res. 2025 Aug 15;10:441. [Version 1] doi: 10.12688/wellcomeopenres.24342.1 (PMC12559846; doi:10.12688/wellcomeopenres.24342.1)
Supplement: Peer review history

# Reviewer report: Reviewer response for version 1

**DOI:** 10.21956/wellcomeopenres.26843.r134305

**Published:** 2025-10-27

**Authors:** Arun Arumugaperumal

**Affiliations:** 1 Department of Biotechnology, Rajalakshmi Engineering College, Thandalam 602105, Chennai, Tamil Nadu, India

The authors have presented the genome sequence of *Psococerastis gibbosa*, a Barkfly. The assembly size reported here is 409.21 Mb, spread among 10 chromosomes. The mitochondrial genome is of size 17.08 kb. The authors have identified 17,010 genes in the fly with the help of Ensembl. The authors have used a male specimen and they are successful in capturing the X,Y sex chromosomes. The authors have used the standard protocols usually followed by the Darwin Tree of Life project. The N50 values greater than the benchmark value indicate that the genome assembly is of good quality. The BUSCO completeness of 97.9% indicates that the genome is complete with respect to sequences in the databases. The background section could have been written elaborately to add more details about the insect. The article can be indexed.

Are sufficient details of methods and materials provided to allow replication by others?

Yes

Is the rationale for creating the dataset(s) clearly described?

Yes

Are the datasets clearly presented in a useable and accessible format?

Yes

Are the protocols appropriate and is the work technically sound?

Yes

Reviewer Expertise:

Bioinformatics; Genomics

I confirm that I have read this submission and believe that I have an appropriate level of expertise to confirm that it is of an acceptable scientific standard.

# Reviewer report: Reviewer response for version 1

**DOI:** 10.21956/wellcomeopenres.26843.r131282

**Published:** 2025-09-23

**Authors:** Panagiotis Ioannidis

**Affiliations:** 1 Foundation for Research & Technology - Hellas, Crete, Greece

This paper describes the sequencing, assembly, and gene annotation of Psococerastis gibbosa, an insect belonging to the Psocodea order. Psocodea is a rather small but ecologically interesting order since it contains lice species. Thus, having more high quality genomes from this order enables even more comparative studies in insects.

 As always, the methodology used in the paper is the standard one used for all DToL assemblies, which results in high quality, chromosome-level assemblies that are suitable for such comparative studies.

 My only criticism has to do with the predicted gene set. Time and again I have mentioned (as a comment in other reviews for this journal) that gene sets MUST be tested with BUSCO in exactly the same way genome assemblies are. Simply giving 17K genes doesn't say much. How good is this gene set? Is it missing conserved genes? Is it missing genes that it shouldn't be missing? These basic questions can be answered with a BUSCO run. You will be surprised how many times a gene set can be worse than it should! And this information should be passed to the user of the genome!

Are sufficient details of methods and materials provided to allow replication by others?

Yes

Is the rationale for creating the dataset(s) clearly described?

Yes

Are the datasets clearly presented in a useable and accessible format?

Yes

Are the protocols appropriate and is the work technically sound?

Yes

Reviewer Expertise:

insect genomics, bioinformatics

I confirm that I have read this submission and believe that I have an appropriate level of expertise to confirm that it is of an acceptable scientific standard.
